# An Experimental Study on Condition Diagnosis for Thrust Bearings in Oscillating Water Column Type Wave Power Systems

**DOI:** 10.3390/s21020457

**Published:** 2021-01-11

**Authors:** Tae-Wook Kim, Jaewon Oh, Cheonhong Min, Se-Yun Hwang, Min-Seok Kim, Jang-Hyun Lee

**Affiliations:** 1 Offshore Industries R&BD Center, Korea Research Institute of Ships & Ocean Engineering (KRISO), 1350 Geojebuk-ro, Jangmok-myeon, Gyeongsangnam-do, Geoje-si 53201, Korea; twook@kriso.re.kr (T.-W.K.); herotaker@kriso.re.kr (J.O.); 2 Research Institute of Industrial Technology, INHA University, 100 Inha-ro, Michuhol-gu, Incheon 22212, Korea; seyun.hwang@gmail.com; 3 Department of Naval Architecture and Ocean Engineering, INHA University, 100 Inha-ro, Michuhol-gu, Incheon 22212, Korea; rhiho1008@nate.com (M.-S.K.); jh_lee@inha.ac.kr (J.-H.L.)

**Keywords:** wave power system, oscillating water column type wave power system, thrust bearing, fault reproduction, fault diagnosis, FMEA, vibration spectrum, machine learning algorithm

## Abstract

In order to utilize wave energy, various wave power systems are being actively researched and developed and interest in them is increasing. To maximize the operational efficiency, it is very important to monitor and maintain the fault of components of the system. In recent years, interest in the management cost, high reliability and facility utilization of such systems has increased. In this regard, fault diagnosis technology including fault factor analysis and fault reproduction is drawing attention as an important main technology. Therefore, in this study, to reproduce and monitor the faults of a wave power system, firstly, the failure mode of the system was analyzed using FMEA analysis. Secondly, according to the derived failure mode and effect, the thrust bearing was selected as a target for fault reproduction and a test equipment bench was constructed. Finally, with the vibration data obtained by conducting the tests, the vibration spectrum was analyzed to extract the features of the data for each operating status; the data was classified by applying the three machine learning algorithms: naïve Bayes (NB), k-nearest neighbor (k-NN), and multi-layer perceptron (MLP). The criteria for determining the fault were derived. It is estimated that a more efficient fault diagnosis is possible by using the standard and fault monitoring method of this study.

## 1. Introduction

In response to the Paris Agreement in 2015, IMO adopted a strategy to reduce the emission of GHG (greenhouse gas) and carbon dioxide in 2018. Korea also announced a significant reduction pledge of about 40% of the GHG emission forecast (GHG emission forecast, business, as usual, BAU) by 2030 [1]. In addition, it aims to reduce GHG by 70% by 2050 to enter the era of decarbonization for solving the emission of carbon dioxide, the ultimate cause of environmental pollution, within this century [2]. As a result, the interest in carbon-free and pollution-free new and renewable energy is greatly increasing. The decarbonization of energy may be achieved when new and renewable energies become the main energy sources.

Among the renewable energy sources, wave energy is one of the energy sources that is receiving much attention because it has high consistency in terms of energy acquisition compared to other energy sources such as wind or solar energy [3]. The wave energy resource available from the world’s oceans is about 2000 TWh per year which theoretically can satisfy the total global power demand [4]. To realize these benefits, the technology for converting waves into useful energy forms was first patented in 1799 [5]. Since then, more than 1000 patents on various concepts have been issued for technology using wave energy [6], and until recently numerous studies on wave power systems have been actively conducted [7,8,9,10,11,12].

**Figure 1:**
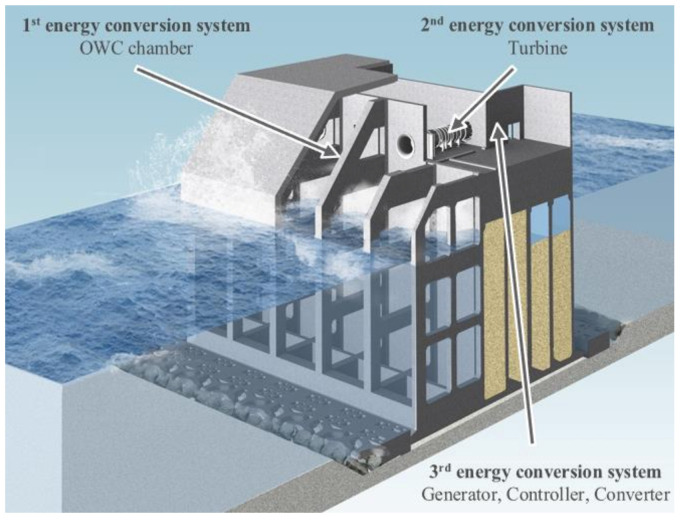
Conceptual diagram of OWC [13].

Among the wave power system, the oscillating water column (OWC) wave power system, shown in Figure 1, represent the primary energy conversion system for converting wave energy into the vertical motion of the wave column inside the chamber, the secondary energy conversion system for rotating the turbine using the airflow generated during the vertical motion of the water column and the final energy conversion system for generating the magnetic field using the rotation of the turbine [13]. This system has a stability advantage over other wave power systems because the mechanical hydraulics and electrical components are not submerged in seawater [14].

Since the wave power system is operated in an environment where severe external forces are applied due to the waves, excessive vibration may be generated in the rotating body resulting in damage to the bearing or turbine blade. Further, due to the damage caused by pressure fluctuation of the air flowing into the turbine, measures to prevent faults must be considered, comprehensively reviewed and reflected in the design. In recent years, the interest in the efficient monitoring and fault diagnosis for such systems has increased greatly [15]. Prognostics and health management (PHM) includes steps of real-time condition monitoring, big-data processing for detected data and the diagnostics and prognostics. Regarding the technology, it comprises technologies for the acquisition of the data for monitoring the condition of the machine using sensors and for diagnosis of the status and prognosis of the remaining lifetime. Through this, it is possible to improve the reliability and operation efficiency [16].

Power generation system Faults are mainly diagnosed by extracting and analyzing the output signals or vibration signals of the system using various mathematical techniques. There are many studies on fault reproduction and diagnosis with abnormal signal processing techniques using deep learning or machine learning algorithms for power generation systems such as wave turbine generators and wind turbine generators [17,18,19,20,21,22]. Furthermore, the needs for fault prediction and residual life prediction technology in wave power generation systems have increased [23,24]. In particular, in this study, the thrust bearing was selected as the target of fault diagnosis among the components of the wave power system through FMEA analysis, and in this regard, research on bearing fault diagnosis using deep learning or machine learning has been very actively conducted in recent years [25,26,27,28,29]. 

**Figure 2:**
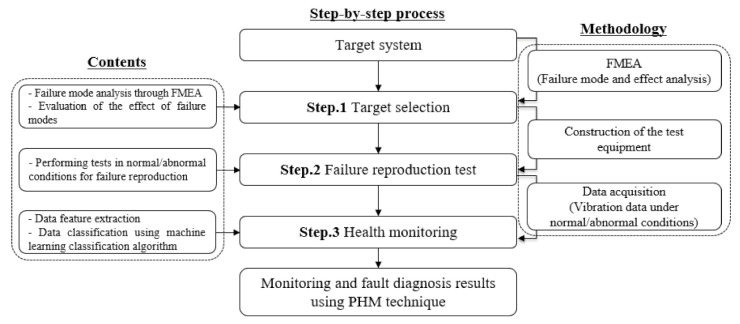
PHM procedure for fault diagnosis system.

Accordingly, in this paper, a study on fault diagnosis was conducted for an oscillating water column type wave power system. Henceforth, after selecting the fault reproducing target, the failure mode of the target was analyzed, and a process for verification using the test equipment and monitoring was presented. Figure 2 shows the PHM process for system fault diagnosis including FMEA provided in this study and construction of suitable test equipment and health monitoring. According to this process, at first, the failure mode of the system is analyzed using FMEA analysis, and the influence of the fault factor is evaluated. Next, the overhaul method is used to check the actual fault of the thrust bearing which is the target selected through FMEA, and a fault reproduction test equipment setup was constructed. Based on this, a test is performed to obtain the vibration data under both normal and abnormal conditions. Finally, the vibration frequency is visualized and the vibration spectrum is analyzed using FFT with the measured vibration sensor data to extract the features of data for each operating condition. Furthermore, the data is classified by applying a machine learning algorithm, and fault diagnosis are performed in accordance with the status monitoring result.

In this study, a new type of test equipment was devised for the unique field of wave power system, the subject of this study, and analysis techniques using machine learning was applied to verify whether the fault diagnosis process and technique for thrust bearings using this test equipment were meaningful. As a result, it is considered that the results obtained through the conducted experimental studies are meaningful and have sufficient correlation with the application to actual structures. Also, it is judged that the new type of test equipment can be used to perform research on reproducing and diagnosing faults for more diverse bearings in the future. 

## 2. Strategy of Diagnosis of Bearing Condition 

### 2.1. Target Selection Using Failure Mode and Effects Analysis (FMEA)

FMEA, when used for identifying and analyzing the potential faults of the products or the processes and their effects, is a method for structuring the risks that may be caused by fault and then preventing the fault in advance [30,31]. 

The turbine system, representative equipment of an oscillating water column type wave power system, comprises a rotor/stator for generating electric power, a blade for converting air flow into mechanical rotational energy, and a shaft supporting various devices and variable bearings. The bearing is applied to reduce damage to the devices by supporting the load generated by the air-flow and the self-weight and inertia of the heavy turbine when rotating the turbine to generate the electric power. The applied bearings include ball bearing, thrust bearing, and NU bearing. The ball bearing and the NU bearing support the vertical load. The thrust beading is installed with several rollers inclined to support vertical and axial loads. 

In this study, FMEA was conducted on these parts, and detailed procedures for FMEA have been replaced by reference [32]. As a result of FMEA, the major failed parts were the turbine blade, slip ring, thrust bearing, and shaft. The turbine blades were recommended as the design change items, the slip rings were recommended as the reliability-based maintenance items, and the shafts were recommended as the real-time monitoring items. In particular, the thrust bearing is the item that cannot be inspected with the naked eye, and it is a component that requires sensor-based monitoring as it causes great damages to the system when the rotational performance gets deteriorated and malfunction occurs. Finally, in this study, the thrust bearing was selected as the target of fault reproduction in accordance with the FMEA results.

### 2.2. Analysis of Thrust Bearing Failure Modes

In order to analyze the fault mechanism and operation status of the thrust bearing, the equipment for fault reproduction selected by FMEA, the degree of wear of the target equipment were analyzed by overhauling the turbine system of the oscillating water column type wave power system located in Yongsu-ri, Jeju-Do. 

**Figure 3:**
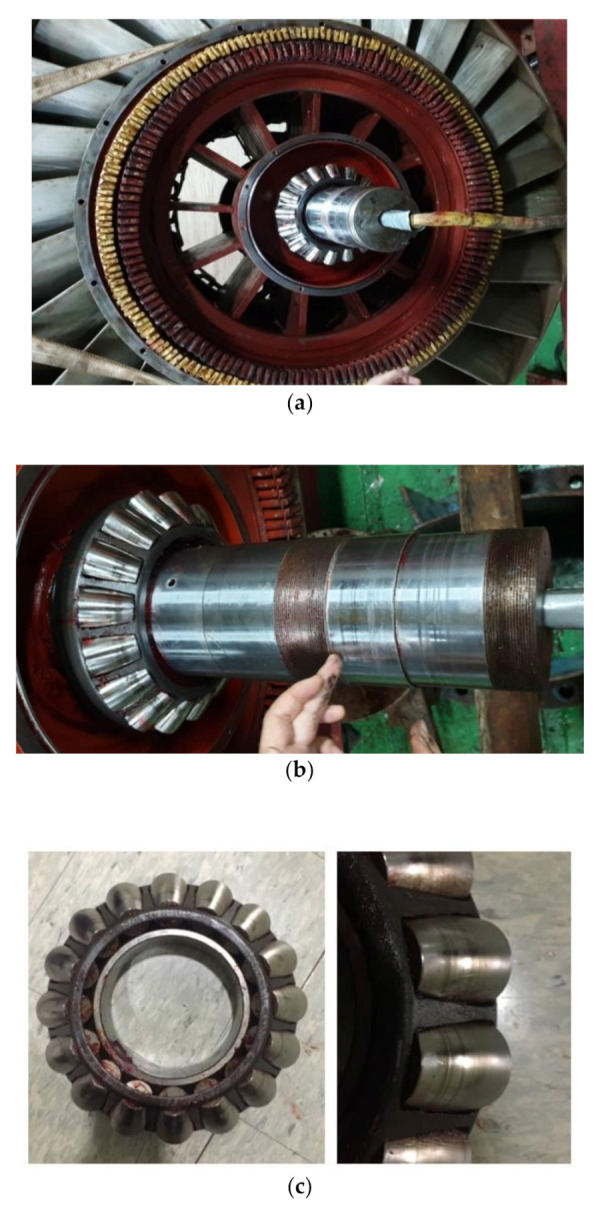
Overhaul results: (**a**) Stator/Rotor; (**b**) Shaft; (**c**) Thrust bearing housing/roller.

As a result of the overhaul (Figure 3), it was confirmed that the thrust bearing was pressed by the load and the wear was caused due to the occurrence of the frictional force by the change in lubricant characteristics during rotation. The wear pattern of the bearing roller occurred in the form of the gradual movement of the wear position in the form of a line instead of the entire surface being worn.

As a result of FMEA and overhaul as mentioned above, it was analyzed that the rotational performance deterioration and the rotation inability were the main factors behind the fault of the wave power system. Bearings were damaged, typically due to 12 types of fault causes including excessive load, overheating, wear, deformation, corrosion, lubricant contamination, and improper assembly [33]. Among them, the causes that affected the rotational performance deterioration and the rotational inability of the thrust bearing were false brinelling, overheating, lubricant fault, and contamination caused by deformation and abrasion on the surface due to the vibration in accordance with the increased surface friction caused by the lubricant contamination and improper lubrication.

### 2.3. Construction of the Test Equipment

Since the thrust bearing is installed inside the turbine, visual inspection is impossible. Accordingly, in order to ensure the structural safety of the thrust bearing, the technology for the prediction of the fault must be applied at the operation stage. In the field of abnormalities and fault conditions of bearings, the researches have been conducted for the determination of the state after fault and analysis of fault conditions from the computational analysis [34,35], etc. In the field of bearing fault diagnosis and prognostics, studies have been conducted for fault diagnosis based on the comparison of the experimental data and the physical models [36], state investigation based on the detected signal analysis [37], and the diagnosis technology in accordance with fault simulation test data [38], etc. Finally, in order to diagnose and prognose the abnormal state of the bearing, it is necessary to obtain the result or data of the abnormal or the fault condition. To do this, it is necessary to be able to reproduce the fault and use the acquired data of the fault state to determine the fault in a reverse manner.

However, it is very difficult to acquire data after the abnormalities or faults in actual equipment. Therefore, there is a need for a method of obtaining data by reproducing operation states of bearing. Most of the previous studies [39,40,41] have reported test equipment in which the inner bearing rotates. However, in this study, we developed a test equipment in which the outer bearing rotates, to reproduce and analyze the phenomena that cause thrust bearing faults.

**Figure 4:**
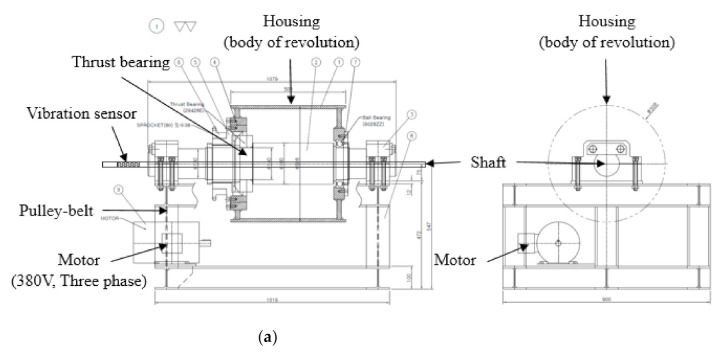

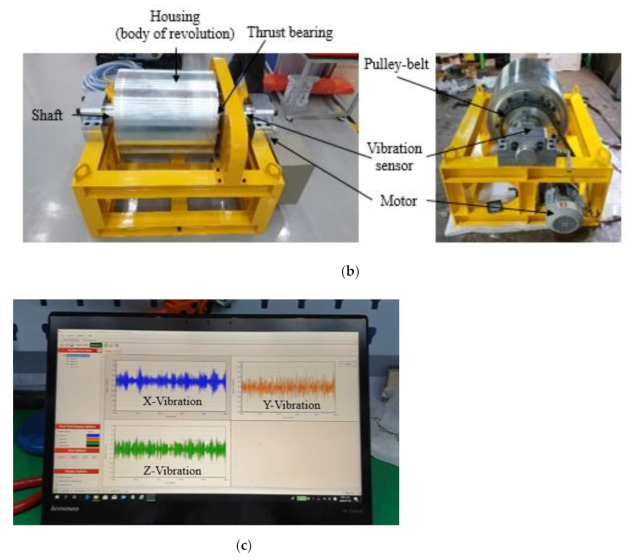
Configurations of test equipment of thrust bearing for simulating the operating condition: (**a**) Design drawing; (**b**) Real appearance; (**c**) Measuring scene.

The developed test equipment (700 kg) comprises a motor, a shaft, a bearing, and a housing. The inner diameter of the rotating body is 140 mm to which the 29428E standard thrust bearing used in the oscillating water column type wave power system can be attached. The design drawing, real appearance and measuring scene of the test equipment are shown in Figure 4. This test equipment is configured as a mechanism that applies a load to the thrust bearing by rotation of the outer ring and uses a motor (60 Hz, 3-phase) and a chain-gear system to rotate the outer ring. The inner ring is fixed to the shaft so that it can support a sufficient load. Also, by adjusting the gear ratio to 30:38 to satisfy the power generation rotational speed of 400 rpm of the oscillating water column type wave power system, it is possible to rotate up to 474 rpm.

To ensure the data according to the normal and abnormal operating conditions of the thrust bearing, a vibration sensor (3-axis) was attached to the non-rotating shaft near the bearing. Data were acquired and stored in real-time through DAQ via a wired vibration sensor, and the measurement was conducted for 1 hour. During the test, the vibration data was measured at intervals of 0.005 seconds at a sampling frequency of 200 Hz.

As mentioned above, bearings are intermediate parts that receive the load of the rotating shaft and transmit them to the support, so lubrication is necessary to reduce the contact area between the ball and the lace. Therefore, in this study, the abnormal operation of the thrust bearing was defined as the false brinelling condition, which is the most important factor for reducing the rotational performance of the bearing as a result of the fault factor analysis. To simulate it, a test was conducted for the abnormal operation condition with a greaseless state. Consequently, the tests were conducted for normal and abnormal operating conditions at 400 rpm which is the actual maximum power generation rpm of the system.

### 2.4. Procedure for Health Monitoring using Classification Algorithms

**Figure 5:**
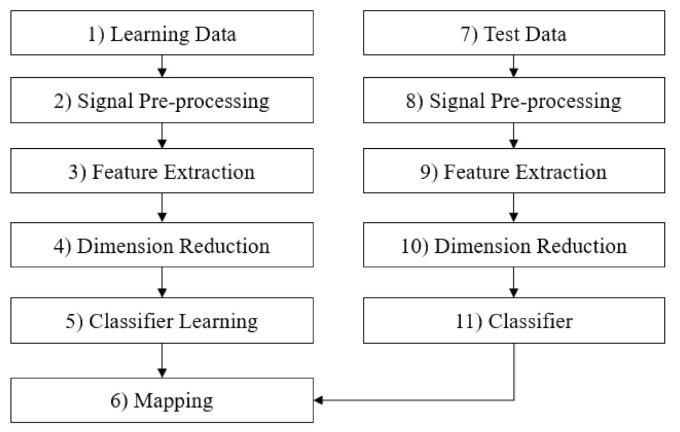
The procedure of fault diagnostics.

This section describes the specific methods for fault diagnosis applied in this study. The fault diagnosis considered in this case study focused on identifying faults determining the failure mode. Figure 5 shows the detailed procedure for fault diagnostics applied in this study. In the first step of fault diagnostics, it is necessary to obtain the fault signal data. In general, we can gather time series data from several sensors attached to test equipment during its operation. In the second step, it is necessary to identify the sensor signal as to whether its data is gathered from the normal or abnormal state with the information on equipment failure, i.e., type of normal state and failure states. 

For the next step, we can extract statistical features in the time and frequency domains from the sensor data by signal pre-processing. Then, from among the selected signal features, a feature that correlates between steady-state and abnormal state and a certain level or more is selected and dimensionally reduced to refine it into 2-dimensional perceptible data. In this process, we reduce the dimension of the features, and build the fault classification map represented by the features through several statistical methods, e.g., principal component analysis (PCA) and the classification algorithms. Then, in the classifier learning process, previous fault history information and normal operation status information are used as the learning data of the fault classifier in the supervised learning scheme. This process creates a classification map for fault diagnosis, i.e., a condition diagnosis criteria.

In this way, the process of extracting factors from the sensor data to indicate the state of the thrust bearing is constructed and determining the state. Therefore, when there is new sensor data, it distinguishes whether it is a normal signal or a fault signal through learned classifier and fault classification map. In case of a fault signal, it is possible to judge which fault type is the fault type. The more detailed procedure is described in the following section.

## 3. Strategy of Diagnosis of Bearing Condition 

### 3.1. Sensor Signal Acquisition 

**Figure 6:**
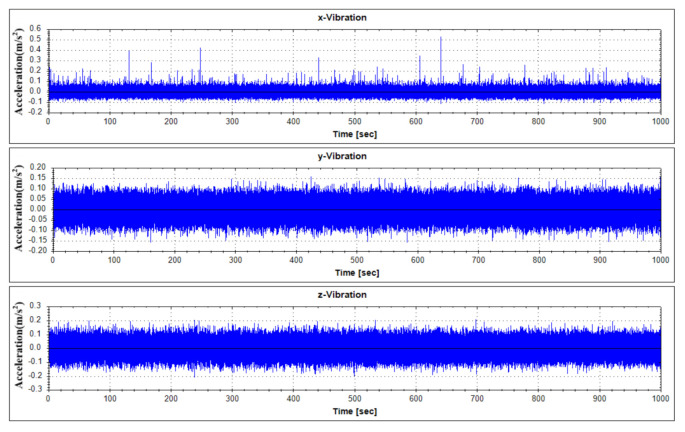
Vibration data history under normal operating states (Mode 1).

**Figure 7:**
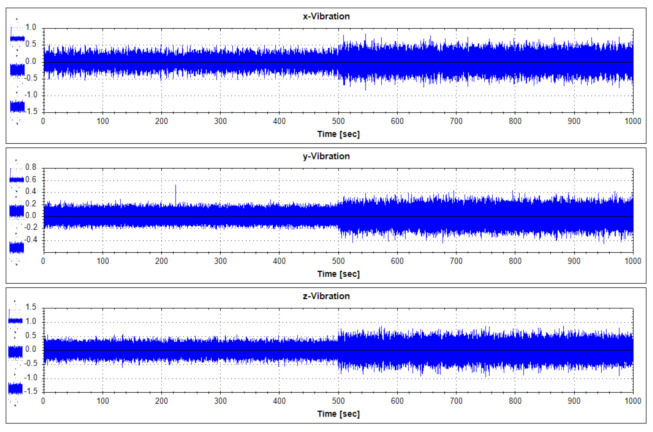
Vibration data history under abnormal operating states (Mode 3).

For fault diagnostics of the equipment, its sensor data that shows the characteristics of fault condition and normal operation condition occurring in the thrust bearing is required. In order to analyze the data generated by the target equipment, the following Table 1 summarizes the operation conditions and obtains the sensor data. Each vibration data according to operation condition was measured by 1000 seconds. Vibration data were measured using a three-axis vibration sensor with components of the x, y, and z axes. Actually, real-time data is measured by attaching a three-axis vibration sensor to a real generator model in Yongsu-ri, Jeju-Do. After the verification of the fault diagnosis technique using the test equipment in this study, we plan to apply it to real-time data. The measured data are representative visualizations of normal state (Mode 1) and abnormal state (Mode 3) as in Figure 6 and Figure 7. For reference, the abnormal operation condition of the thrust bearing defined in this study is a greaseless state. In the experiment under this condition, it was observed that the vibration response became very large and the bearing temperature rapidly increased.

### 3.2. Feature Extraction

For the next task, it is necessary to extract the features that represent the characteristics of the vibration sensor signal in a proper way in terms of status monitoring. To this end, in this study, the feature extraction scheme based on statistical values is applied in the frequency and time domains. It is not reasonable to classify time–frequency distributions directly because the data dimensions are too high to deal with. Thus, the statistical values are utilized to reduce the high dimensionality feature space. To minimize the number of features used and the classification error, we can use the features generally used in analyzing the failure. For example, those of the rotatory machine are described in Table 2 and Table 3. Features extracted from the time domain of the time-series sensor signal and Features extracted from the frequency domain were selected. Note that X is original sensor data and μ is the mean value of the sensor data in the tables [16]. 

**Figure 8:**
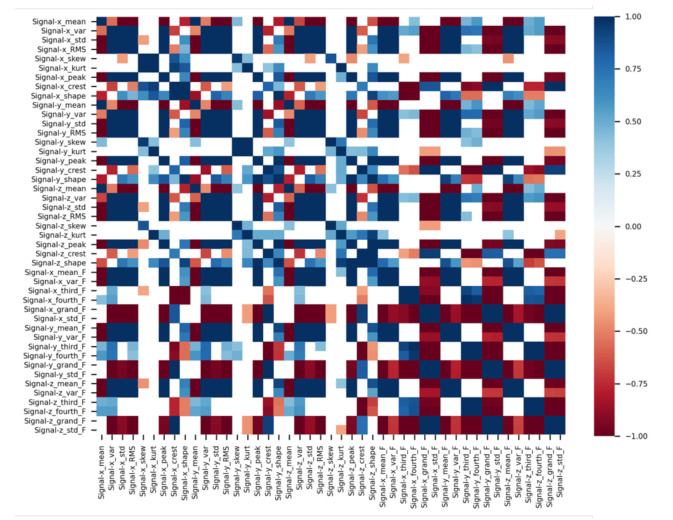
Heat map through correlation analysis of selected features.

In such a case, it may be difficult to select a sensor list to determine the type of fault, and it may be necessary to manage unnecessary data that is not related to the fault diagnostics. In this case, it may be efficient to configure and manage one unit according to the operating states. Therefore, a list of sensors that can represent the status of the target system should be appropriately selected, excluding the unnecessary ones among the sensors included in the equipment. To this end, in this study, the Pearson correlation coefficient between each sensor signal is calculated using Equation (1) to classify the sensor list having a significant correlation, and only signals of the target sensors are acquired. Extracting the 15 factors to be selected for the three-axis vibration sensor produces a total of 45 features. Analysis of the 45 features extracted by the correlation analysis results in a heat map as in Figure 8. For the feature dependency approach, the correlation coefficient plays an important role though it has not been used as often as mutual information. From the definition of the coefficient, the correlation provides a quantitative measurement that represents the strength of a relationship between sequences of data. In other words, the features that affect the condition monitoring of thrust bearings are applied and features which lower the overall accuracy by the learning machine will be removed from the original feature set. However, the procedure would be progressively repeated until the classification accuracy cannot be further improved. This procedure needs complicated computation and always takes a lot of time [42]. Therefore, in this study, appropriate values were selected through some case study until the accuracy that affects classification acuity was found. The correlation coefficient chosen through case study is 50%, which is applied to the machine learning algorithm:

(1) $$\rho_{x,y}=\frac{cov\left(x,y\right)}{\sigma_{x}\sigma_{y}}$$

(2) $$cov\left(x,y\right)=E\left[\left(x-\mu_{x}\right)\left(y-\mu_{y}\right)\right]$$

where, $\rho_{x,y}$ is the Pearson correlation coefficient of the x, y signal matrix, and σ and μ are the standard deviation and the mean value, respectively. Also, $cov\left(x,y\right)$ is a covariance matrix as shown in Equation (2).

### 3.3. Dimensionality Reduction 

**Figure 9:**
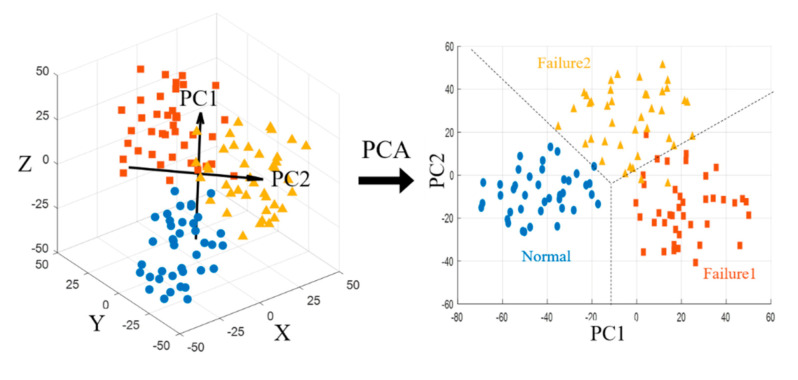
Mapping of features on two—dimensional space through principal component analysis.

To simplify the features of sensor data having a high-dimensionality vector form, a process of reducing the dimensions is required. Dimensionality reduction in data processing focuses on representing data with the minimum number of dimensions such that its properties are not lost and hence reducing the underlying complexity in processing the data. PCA is one of prominent dimensionality reduction techniques. In this study, to minimize the dimensionality of the features and classify the fault condition clearly, two principal components having the high eigen values are selected as shown in Figure 9. The principal components can be represented by the following Equation (3).

(3) $$PC_{i}=a_{1}X_{1}+a_{2}X_{2}+\cdots +a_{n}X_{n}$$

where $PC_{i}$ denotes the principal component i, $X_{n}$ and $a_{n}$ represent the original feature n and numerical coefficient for $X_{n}$, respectively. 

**Figure 10:**
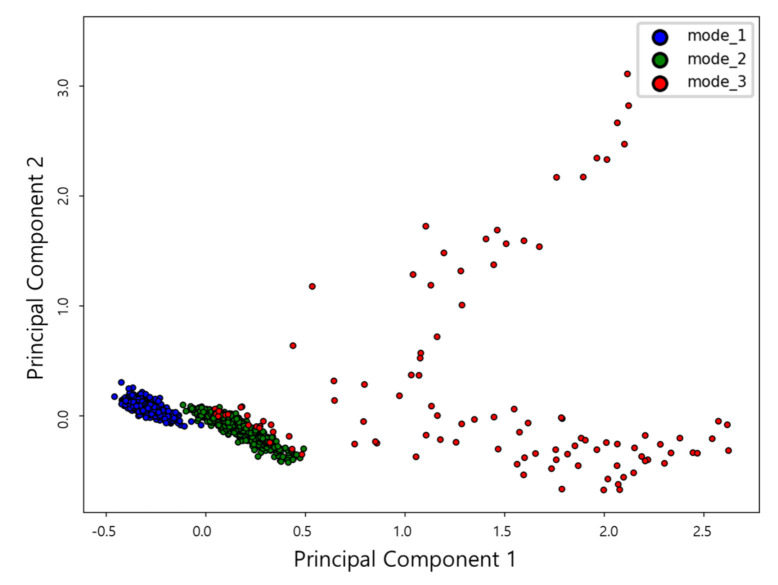
Mapping of features on two—dimensional space through principal component analysis.

The results of the dimension reduction of selected features in two dimensions through correlation analysis and PCA analysis are show in Figure 10. It is possible to verify that each data set is clustered in 2-dimensional map for each mode 1, 2 and 3 of the conditions of the thrust bearing.

### 3.4. Classifier Learning

After identifying the suitable features, in order to classify the sensor data patterned according to the state of each signal, we need a classification process with a suitable classifier. The classifier is a criterion for distinguishing between each type of features of sensor data expressed in two dimensions space. The classification process recognizes the given data into a suitable pattern for the features based on the criteria obtained by learning the sensor data of the existing fault and normal operating state. The applied classification model are representative classifier algorithms commonly used for health monitoring. In order to learn the operation status classification model using the refined dataset shown in Figure 10, major parameters that can determine performance for each algorithm shall be established. Therefore, how the parameters are set for each algorithm determines the accuracy of the model. In this work, we conduct a study to select parameters to determine appropriate classification boundaries for the following three algorithms: naïve Bayes (NB), k-nearest neighbor (k-NN), and multi-layer perceptron (MLP). The refined data features are each determined by the classification algorithms.

#### 3.4.1. Naïve Bayes (NB)

Naïve Bayesian classifiers assign the most likely class to a given example described by its feature vector expressed in Equations (4) and (5). They determine the point where the error is minimized by appropriately moving the decision boundary and finding the minimum error where the probability density function of two data classes is idealized. Therefore, the features can be simplified by assuming that features are in an independent class for fault diagnostics. Naïve Bayes can be divided into Gaussian naïve Bayes, Bernouli naïve Bayes and multinolomic naïve Bayes, depending on the classification model, and in this work, the distribution of data is visually well classified and applied the Gaussian classification model.

(4) $$P\left(x\left|y\right.\right)=\frac{P\left(x\cap y\right)}{P\left(y\right)}=\frac{P\left(y\left|x\right.\right)P\left(x\right)}{P\left(y\right)}$$

(5) $$\overset{\wedge }{y}=\underset{k\in \left\{1,\cdots k\right\}}{argmax}p\left(C_{k}\right)\prod_{i=1}^{n}p\left(x_{i}\left|C_{k}\right.\right)$$

##### 3.4.2. k-Nearest Neighbor (k-NN)

**Figure 11:**
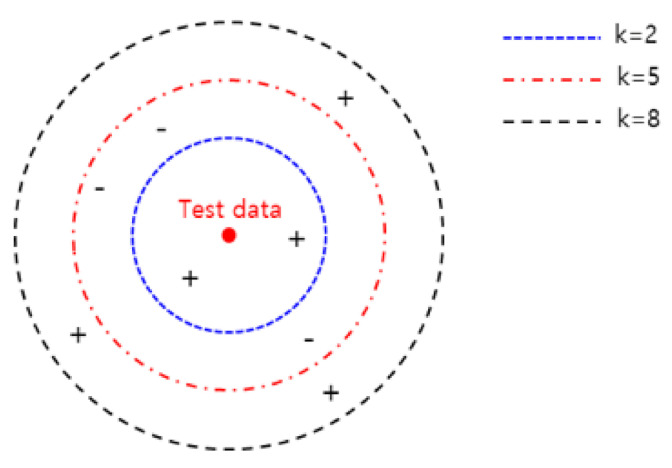
The diagram of k-NN.

k-NN is an instance-based learning algorithm that determines how closely an instance is located with other objects with similar characteristics [43]. For example, the characteristics of a red dot, such as Figure 11, can be inferred from the surrounding neighbors. If k is 2, 5, 8, the red dot is classified as + and k is 3. where k is the number of objects closest to the red dot. As a major parameter of k-NN, the distance between the data, representing the number of neighbors, is applied to the Minkowski distance method.

#### 3.4.3. Multi-Layer Perceptron (MLP)

MLP is a representative artificial neural network model consisting of three layers: input layer, hidden layer, and output layer, as shown in Equation (6) [44]. The neurons in the MLP are defined as input $x_{i}$ and output y. f is the activation function, and the sigmoid function is generally used. $W_{i}$ is the MLP model weight vector, and b is the slice (bias) value. The weights are calculated by repeated optimization procedures based on input and output learning data.

The main parameter of MLP is an activation function that converts the input value of hidden layers to nonlinear. In this work, 100 hidden layers were selected, auto batch size selection methods were applied to determine how much training data is partitioned and learned, and the Adam solver was applied to calculate the optimized weights.

(6) $$y=f\left(W^{T}x\right)=f\left(\sum_{i=1}^{3}W_{i}x_{i}+b\right)$$

## 4. Results of Machine Learning using the Classification Algorithms 

### 4.1. Learning Results of the Classification Algorithms

**Figure 12:**
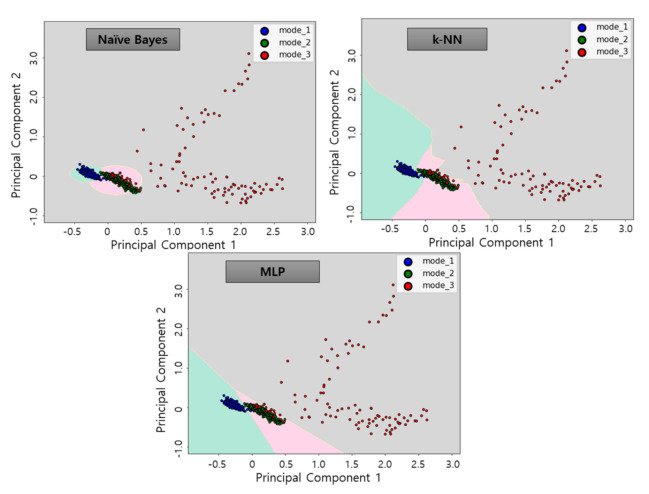
Learning results using classification algorithms by applying learning data.

The 45D features extracted was screened by correlation analysis and dimensioned to 2D by applying PCA algorithm. Subsequently, three classification algorithms allow the creation of classification models to display decision boundaries as in Figure 12. By using the classification algorithms, the decision boundaries were selected for each data group as follow each mode. As shown in Figure 12, the operation status was determined in three states: Mode 1 (blue points—green background), Mode 2 (green points—red background), and Mode 3 (red points—gray background). The boundaries, or background colors, determined in this process, is the decision boundary plane determined by each algorithm and refers to the learning model. These learning model represents the operation status of each of the operations described in Table 1. In order to avoid overfitting in the learning process using the classification algorithms, the selected learning data were randomly selected 80% of the learning data set with 20% of the test data. The learning accuracy for each algorithm was 98.1% for NB, 99.1% for k-NN and 99.1% for MLP, with high accuracy calculated for all algorithms.

### 4.2. Verification of the Classification Algorithms

**Figure 13:**
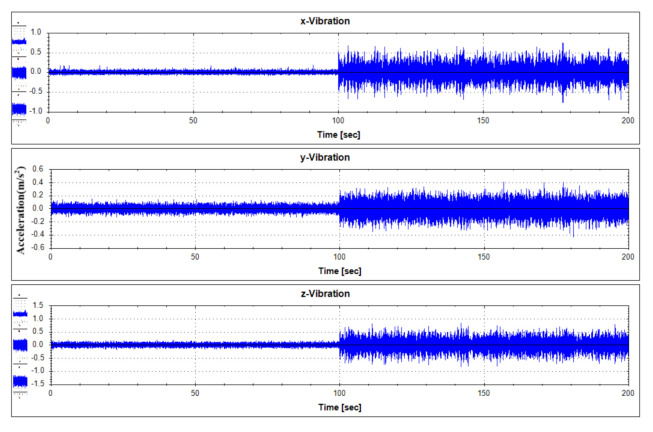
Vibration data history of test data set No. 1.

**Figure 14:**
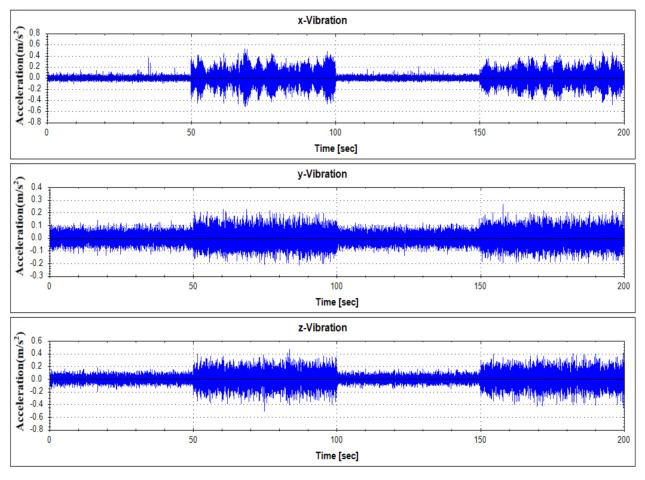
Vibration data history of test data set No. 2.

**Figure 15:**
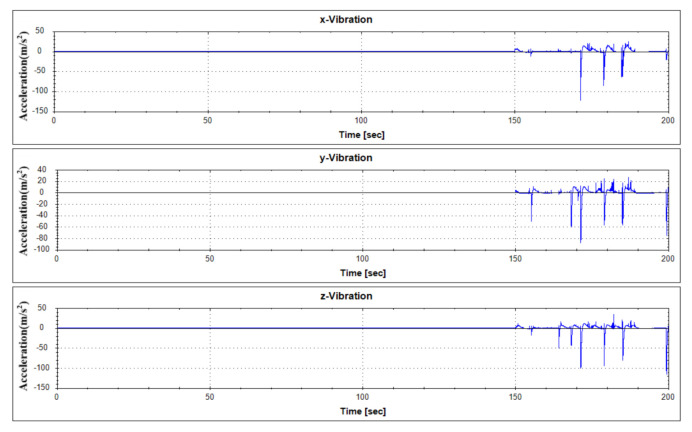
Vibration data history of test data set No. 3.

Each classifier algorithm was verified by applying different data from the learning data to identify the proposed fault diagnosis procedure. The applied test data consisted of three data sets, combining data from normal state and abnormal state 1 and 2, as shown in Table 4. The vibration data history of the applied test data set is as shown in Figure 13, Figure 14 and Figure 15.

**Figure 16:**
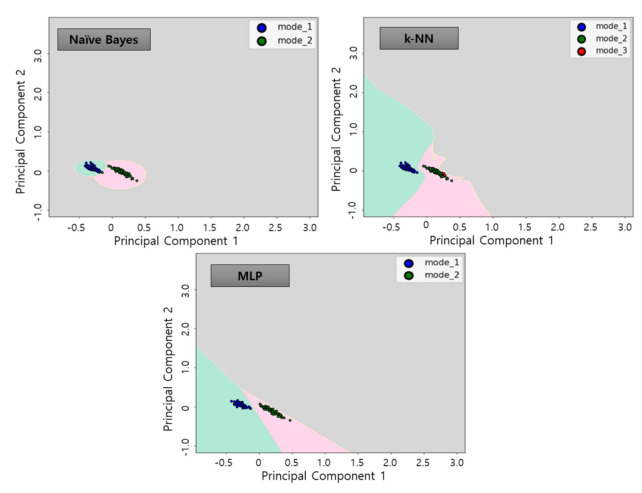
Results of classifier algorithm application of test data No. 1.

**Figure 17:**
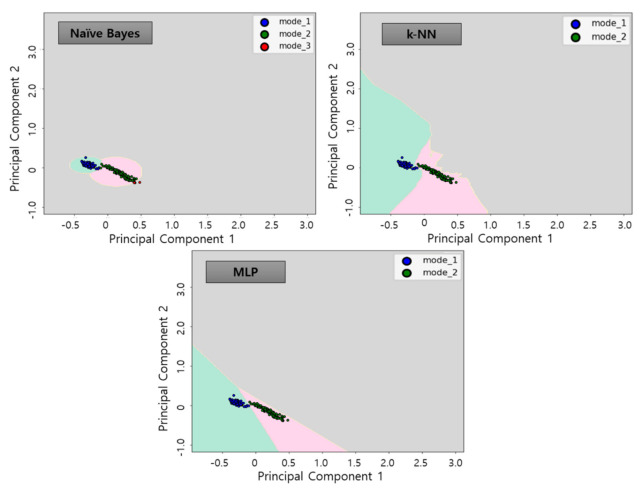
Results of classifier algorithm application of test data No. 2.

**Figure 18:**
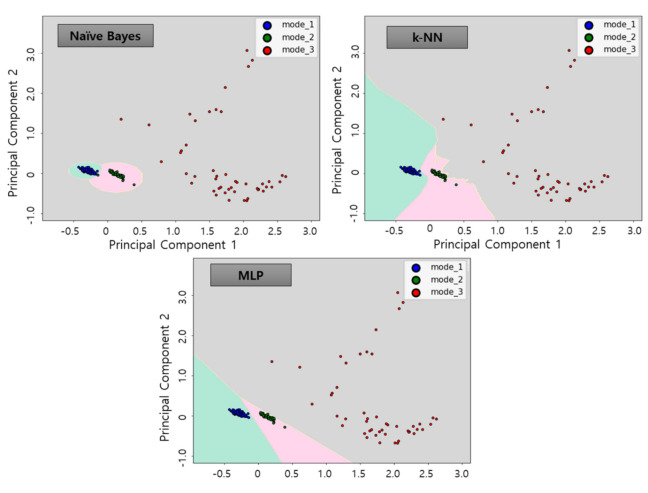
Results of classifier algorithm application of test data No. 3.

The diagnosis using the three classifier algorithms proposed in this study by applying the test data sets resulted in a high accuracy of about 99% for all algorithms, as in Table 5. Figure 16, Figure 17 and Figure 18 show the results of applying test data for each algorithm.

## 5. Conclusions

The present work describes a study on the diagnosis of bearing condition from the failure mode effect analysis and the test for the fault reproduction and monitoring of the oscillating water column type wave power system installed on the sea of Jeju-do. As a result of the analysis of the failure mode of the wave power system, it was derived that the thrust bearing was essential equipment for monitoring the target, and in particular, the false brinelling characteristics were analyzed as the main management target among several fault factors. For the diagnosis of faults of the thrust bearing, data on the actual faults are required. Therefore, a test equipment setup was built and an experiment was conducted for fault reproduction for the thrust bearing. The main results are summarized as follows:

- An outer ring rotating test equipment setup was developed that can ensure the abnormal and fault data by reproducing the operation environment of the bearing in use. Using the developed test equipment, the vibration data in the normal operation state and false brinelling state were measured, and the characteristics of the data were extracted by analyzing the vibration spectrum.
- As a result, features were extracted by applying a feature extraction scheme based on statistical values in the frequency and time domains of the vibration data in the normal and abnormal state acquired in the test equipment. At this time, a total of 45 features are created for the three-axis vibration sensor. In this study, features with more than 50% correlation were applied to three classification algorithms: NB, k-NN, MLP.
- The learning accuracy for each algorithm was 98.1% for NB, 99.1% for k-NN and 99.1% for MLP, with high accuracy calculated for all algorithms. By applying the test data sets to the three classifier algorithms proposed in this study, it was confirmed that the accuracy of all algorithms was approximately 99%.
- Finally, the subject of this study is a unique field of wave power system, and its application to this field is considered to be of great value. By accumulating data using the test equipment of this study, this study is considered to be a cornerstone for the failure reproduction of the wave power system. In addition, it is possible to conduct a study to diagnose a failure in connection with a simulation using the accumulated data.

In future research, the acquired data will be developed as a smart monitoring engine through AI learning. Further, a variety of features will be acquired by adding sensors that can analyze various features such as heat, sound, and displacement, as well as vibration sensors. In addition, it is planned to establish a database of fault conditions by additionally measuring the various features from the reproduction of additional abnormalities and the fault conditions including contamination. Finally, the application of the fault diagnosis process and technique of this study to the real data obtained from the actual wave power system is planned to be dealt with in the next study. 

The ultimate purpose of this study is to implement the influencing factors and acquire the synergistic effect by establishing an experimental design method in which all the factors affecting the fault of the thrust bearing is considered. It is estimated that this study can be considered as a basis of bearing fault diagnosis in order to obtain the synergistic effect. By developing these details of the study, it is considered that it will be possible to cost down in the future when designing a rotating body including various bearings.

## Figures and Tables

**Table 1:** List of test conditions in normal and abnormal states.

Mode No.	Operation Condition	Operation RPM	Fault
1	Normal state	400	No fault
2	Abnormal state 1	400	No grease
3	Abnormal state 2	400	No grease + fault

**Table 2:** Feature index used in the test signal in time domain.

Feature Index	Description
Mean	$T1=\frac{\sum_{n=1}^{N}x\left(n\right)}{N}$
Variance	$T2={\left(\frac{\sum_{n=1}^{N}\sqrt{\left|x\left(n\right)\right|}}{N}\right)}^{2}$
Standard deviation	$T3=\sqrt{\frac{\sum_{n=1}^{N}{\left(x\left(n\right)-T1\right)}^{2}}{N-1}}$
RMS	$T4=\sqrt{\frac{\sum_{n=1}^{N}{\left(x\left(n\right)\right)}^{2}}{N}}$
Skewness	$T5=\frac{\sum_{n=1}^{N}{\left(x\left(n\right)-T1\right)}^{3}}{\left(N-1\right)\sigma^{3}}$
Kurtosis	$T6=\frac{\sum_{n=1}^{N}{\left(x\left(n\right)-T1\right)}^{4}}{\left(N-1\right)\sigma^{4}}$
Peak value	$T7=\frac{1}{2}\left[\max\left(x\left(n\right)\right)-\min\left(x\left(n\right)\right)\right]$
Crest factor	$T8=\frac{T7}{T4}$
Shape factor	$T9=\frac{T4}{\frac{\sum_{n=1}^{N}\left|x\left(n\right)\right|}{N}}$

**Table 3:** Feature index used in the test signal in frequency domain.

Feature Index	Description
Mean	$F1=\frac{\sum_{m=1}^{M}y\left(m\right)}{M}$
Variance	$F2=\frac{\sum_{m=1}^{M}{\left(y\left(m\right)-F1\right)}^{2}}{M-1}$
Third moment	$F3=\frac{\sum_{m=1}^{M}{\left(y\left(m\right)-F1\right)}^{3}}{M{\left(\sqrt{F2}\right)}^{3}}$
Fourth moment	$F4=\frac{\sum_{m=1}^{M}{\left(y\left(m\right)-F1\right)}^{4}}{M{\left(F2\right)}^{2}}$
Grand mean	$F5=\frac{\sum_{m=1}^{M}f_{m}y\left(m\right)}{\sum_{m=1}^{M}y\left(m\right)}$
Standard deviation	$F6=\sqrt{\frac{\sum_{m=1}^{M}{\left(f_{m}-F5\right)}^{2}y\left(m\right)}{M}}$

**Table 4:** Test data set for the evaluation of the machine learning algorithms.

Dataset No.	Description	Mode Combination
1	Normal state (100 sec) + Abnormal state 1 (100 sec)	Mode 1 & Mode 2
2	Normal state (50 sec) + Abnormal state 1 (50 sec) + Normal state (50 sec) + Abnormal state 1 (50 sec)	Mode 1 & Mode 2
3	Normal state (100 sec) + Abnormal state 1 (50 sec) + Abnormal state 2 (50 sec)	Mode 1 & Mode 2 & Mode 3

**Table 5:** Accuracy of classification algorithms of test data.

Data Set No.	Naïve Bayes	k-Nearest Neighbor	Multi-Layer Perceptron
1	99.5%	99.0%	100%
2	98.0%	100%	100%
3	99.5%	99.5%	99.5%

## Data Availability

Not applicable.
